# Human stefin B: from its structure, folding, and aggregation to its function in health and disease

**DOI:** 10.3389/fnmol.2022.1009976

**Published:** 2022-10-21

**Authors:** Eva Žerovnik

**Affiliations:** ^1^Department of Biochemistry and Molecular and Structural Biology, Jožef Stefan Institute, Ljubljana, Slovenia; ^2^Jožef Stefan International Postgraduate School, Ljubljana, Slovenia

**Keywords:** progressive myoclonus epilepsy of type 1 (EPM1), cystatin B, autophagy, mitochondrial damage, oxidative stress, proteostasis, oligomeric state, synapse

## Abstract

Mutations in the gene for human stefin B (cystatin B) cause progressive myoclonic epilepsy type 1 (EPM1), a neurodegenerative disorder. The most common change is dodecamer repeats in the promoter region of the gene, though missense and frameshift mutations also appear. Human stefin B primarily acts as a cysteine cathepsin inhibitor, and it also exhibits alternative functions. It plays a protective role against oxidative stress, likely *via* reducing mitochondrial damage and thus generating fewer mitochondrial reactive oxygen species (ROS). Accordingly, lack of stefin B results in increased inflammation and *NLRP3* inflammasome activation, producing more ROS. The protein is cytosolic but also has an important role in the nucleus, where it prevents cleavage of the N terminal part of histone 3 by inhibiting cathepsins L and B and thus regulates transcription and cell cycle. Furthermore, it has been shown that stefin B is oligomeric in cells and that it has a specific role in the physiology of the synapse and in vesicular transport. On the basis of my research team’s data on the structure, folding, and aggregation of stefin B, we have proposed that it might regulate proteostasis, possessing a chaperone-like function. In this review, I synthesize these observations and derive some conclusions on possible sources of EPM1 pathology. The interaction partners of stefin B and other gene mutations leading to EPM1-like pathology are discussed and common pathways are pinpointed.

## Introduction

Epileptogenesis is believed to result from an imbalance of ions in neurons. Various factors can cause an influx of Ca^2+^or an imbalance of K^+^ and Cl^−^ ions and therefore lead to overexcitability of the neurons and to excitotoxicity caused by glutamate release (Cano and Fonseca, 2021). Similarly, mutations in ion channels cause some inherited epilepsies. However, as neurodegeneration and traumatic brain injury are also often accompanied by the development of seizures, it is to be expected that there are some common molecular pathways between epilepsy and major neurodegenerative diseases (Surguchov et al., 2017; Cano and Fonseca, 2021).

Progressive myoclonic epilepsy type 1 (EPM1), also called Unverricht-Lundborg disease, is an autosomal recessive disorder caused by mutations in the cystatin B (CSTB) gene, which acts as a cysteine protease inhibitor (Pennacchio et al., 1996; Lalioti et al., 1997a, b). The protein is called stefin (Brzin et al., 1983), but is also known as cystatin. According to the MEROPS classification, stefins are classified as clan 1A of the cystatins (Rawlings et al., 2018). In contrast to cystatin C, stefin B (cystatin B) is a bit shorter (at 98 amino acids long) and carries only one Cys in the third position from the N-terminus. Various mutations in the stefin B gene have been found in patients with EPM1. Most of them are dodecamer repeats located in the promoter region of the gene, resulting in up to a 95% decrease in stefin B mRNA expression (Lalioti et al., 1997a; Joensuu et al., 2007, 2008).

EPM1 is classified as a neurodegenerative disorder, as a lack of stefin B function leads to neurodegeneration of the Purkinje cells in the cerebellum. The disease presents with stimulus-sensitive myoclonus and tonic-clonic epileptic seizures, which are induced by various stressors such as sound, light, or physical exercise. Symptoms typically start between ages 6 and 17. With the progression of the disease, patients develop many neurological symptoms such as ataxia, intention tremor, and dysarthria. One-third of EPM1 patients become wheelchair users, mostly due to progressive myoclonus and ataxia (Kälviäinen et al., 2008).

In the cytosol, stefin B in its monomeric form acts as a cysteine cathepsin inhibitor and exerts a lysosomal function (Alakurtti et al., 2005). Dimers (Jerala and Žerovnik, 1999; Staniforth et al., 2001) and tetramers (Jenko Kokalj et al., 2007) of stefin B form easily *in vitro* by the mechanism of domain swapping. That stefin B (cystatin B) dimers and oligomers are also present in cells was shown by the Italian research teams led by M. Melli (Cipollini et al., 2008; Rispoli et al., 2013), who recently reported that cystatin B plays a role in the physiology of the synapse and in vesicular transport (Penna et al., 2019). Which of these functions happen upstream is not yet clear. Its nuclear localization and function(s) (Riccio et al., 2001; Čeru et al., 2010a) are undoubtedly important.

As early as 2005, my research team predicted that the neurodegeneration observed in EPM1 may be due, at least in part, to the protein aggregation phenomenon (Čeru et al., 2005). Indeed, different pathological mutants of stefin B have been found, such as frame-shift, missense, and splice mutants, apart from dodecamer repeats (Joensuu et al., 2007). Some of these mutations lead to protein misfolding and aggregation (Čeru et al., 2010b; Polajnar et al., 2012; 2014a), causing a loss of function and thus contributing to a more severe phenotype (Koskenkorva et al., 2011).

On the basis of our data on the protein aggregation of stefin B in cells, we proposed that stefin B might regulate proteostasis, possessing a chaperone-like function (Škerget et al., 2010; Taler-Verčič and Žerovnik, 2010). We believe that more insight into the common pathways with other neurodegenerative diseases and EPM1 can be derived from such alternative functions of stefin B (Žerovnik, 2017a), which are not dependent on the inhibition of the cathepsins. More insight into the cellular mechanisms and pathology of EPM1 can also be gleaned from other gene mutations that cause phenotypes similar to EPM1.

In this review, I attempt to relate the molecular and cellular studies of stefin B to EPM1 pathology. However, until more neuronal and clinical studies are done, my derivations remain hypothetical. The key seems to be understanding the alternative functions of oligomeric stefin B in cells and on membranes, including glia and neuronal cells, with loss of function and/or gain in toxic function by the mutants leading to mitochondrial and autophagy dysfunction and, consequently, an increase in reactive oxygen species (ROS), causing neurodegeneration. Another source of neurodegeneration might be increased oxidative stress from different sources.

## Stefin B: A General Introduction

Under normal physiological conditions, human stefin B (cystatin B), as a housekeeping gene, acts as an intracellular cathepsin inhibitor. In order to understand increased oxidative stress and inflammation due to stefin B deficiency, one must look into the location of this protein, its interactions, and its normal and alternative functions in the cell.

### Gene, protein, localization, and pathology

The gene for human cystatin B (*CSTB*) is located on human chromosome 21 (Hattori et al., 2000). Chromosome 21 is the smallest human chromosome, encoding approximately 252 proteins. Among them are the genes for key proteins related to amyloidogenesis, vascular and synaptic plasticity, and oxygen/peroxide metabolism, such as amyloid precursor protein, beta secretase-2, alpha-crystallin A chain, C21 or f62, *SUMO3*, superoxide dismutase, superoxide dismutase-1 (SOD-1), synaptojanin, and stefin B (cystatin B). Notably, trisomy 21, also known as Down syndrome, is caused by an extra copy of chromosome 21.

Stefin B protein is located in the cytosol, mitochondria, and nucleus of cells (Čeru et al., 2010a). In the nucleus, stefin B deficiency results in histone H3 N-terminus cleavage, exerted by cathepsins L and B (Daura et al., 2021). This cleavage results in significant transcriptional changes of the nuclear-encoded mitochondrial genes (Daura et al., 2021). Our team has previously demonstrated (Čeru et al., 2010a) that stefin B in the nucleus interacts with nucleosomes, specifically with histones H2A.Z, H2B, and H3, as well as cathepsin L.

Stefin B was found multimeric in cells (Cipollini et al., 2008; Rispoli et al., 2013), which suggests it may have alternative functions than cysteine proteases inhibition. Its oligomers were shown to interact with cytoskeletal proteins (Di Giaimo et al., 2002) and to bind Aβ (Škerget et al., 2010), suggesting a chaperone-like function (Taler-Verčič and Žerovnik, 2010; Žerovnik, 2017b). Recently, the Italian researchers have demonstrated that stefin B (cystatin B) is important in physiology of the synapse (Penna et al., 2019) and is essential for proliferation and interneuron migration (Di Matteo et al., 2020). When the cystatin B gene is mutated and either loses activity or aggregates, or both, it causes a rare progressive myoclonic epilepsy—EPM1—that occurs throughout the Baltics and some parts of the Mediterranean (Kälviäinen et al., 2008).

### The structure of stefin B in monomeric and oligomeric forms

**Figure 1:**
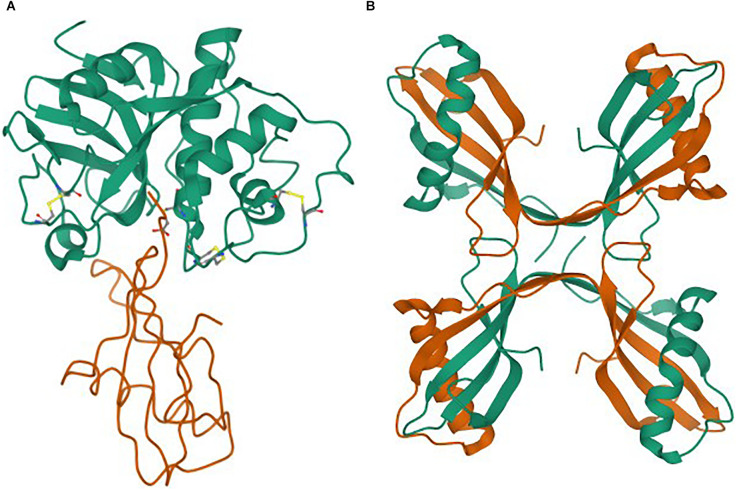
**(A,B)** Structures as taken from the Protein Data Bank (PDB). **(A)** Monomer of stefin B in complex with papain (Stubbs et al., 1990). **(B)** Tetramer of stefin B (Jenko Kokalj et al., 2007).

The monomeric structure of stefin B, in complex with papain, was determined long ago by (Stubbs et al., 1990; as shown in Figure 1A). The structure of stefin B tetramer was determined by (Kokalj et al., 2007; shown in Figure 1B). This small globular protein is composed of a β-sheet of five β-strands and an α-helix. Its stability and folding have been extensively studied, and is described below.

### *In vitro* studies of stability and the folding mechanism

Human stefin B (stB) serves primarily as a good model for amyloidogenic proteins. A number of studies have been performed on the stability and folding kinetics of stefins (Žerovnik et al., 1991, 1992a, b, c, 1998a, b, 1999, Jerala et al., 1998). Major differences have been observed in the stability and folding mechanism between human stefin A and human stefin B (Žerovnik et al., 1991, 1992a, c, 1999, Kenig et al., 2001). It has been shown that stefin A folds reversibly and according to a two-state mechanism, while stefin B populates equilibrium and folding intermediates (Žerovnik et al., 1992a, c, 1997, 1998a, b, 1999; Jerala and Žerovnik, 1999; Kenig et al., 2001). To determine which structural part influences the folding mechanism of the two stefins, we produced their chimera with exchange of α-helices (Kenig et al., 2006). It was confirmed that the α-helix of stefin B adds to the rate of folding, which is about 10-fold faster for stefin B than for stefin A (Kenig et al., 2006). This difference in the rate of folding remained in all chimeras possessing the α-helix of stefin B. The special role of Tyr 31 in stefin B human isomorph was also explored (Jelinska et al., 2011). When this residue was replaced with either Glu (glutamate) present in the more abundant polimorph or Thr (threonine), the rate of folding slowed down to a similar rate as that observed for stefin A. Looking into the stefin A α-helix in comparison to stefin B, Pro25 was identified as a possible interfering residue, which could lead to a kink in the α-helix of stefin A. However, replacing this Pro (proline) with a Ser (serine; did not increase the rate of folding of stefin A (Jelinska et al., 2011). It was concluded that stefins represent an interesting case of proteins that conform to a mechanism somewhere between the framework and the hydrophobic collapse models. In the framework model, the secondary structure forms first, and in the hydrophobic collapse model, an intermediate with a non-native secondary structure (as observed with stefin B α-helix) and an internal hydrophobic core forms (leading to molten globule intermediate, also observed in stefin B folding; Žerovnik et al., 1992c, 1998a, b, 1999).

### Oligomerization of cystatins is governed by domain-swapping

**Figure 2:**
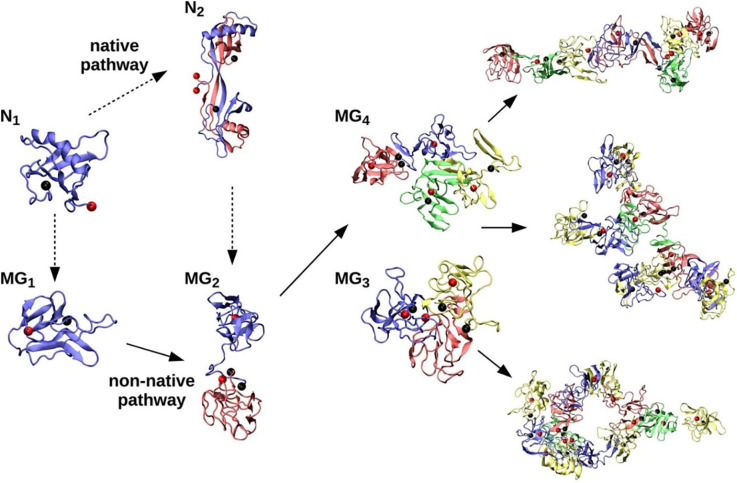
Native (N) and non-native (MG) assembly pathways of stefin B-var2. Under partially denatured conditions, the native monomer (N_1_) forms a stable domain-swapped dimer (N_2_). Native monomers (N_1_) and dimers (N_2_) are denatured (dashed arrows) under amyloidogenic conditions, resulting in MG monomer (MG_1_) and MG dimer (MG_2_) populations that further assemble into MG trimers (MG_3_), MG tetramers (MG_4_), and larger oligomers of various morphologies (elongated octamer, branched undecamer, and annular dodecamer, from top right to bottom right, respectively). Reproduced from Zganec et al. (2015).

Structure and folding studies have shown that stefins, including the more stable stefin A, might form oligomers of domain-swapped type (Jerala and Žerovnik, 1999). When a tetramer of stefin B was crystallized, loops of two domain-swapped dimers over twin were observed, involving proline isomerism of Pro74, which is initially in a *cis* isomer (Jenko Kokalj et al., 2007). Previously, domain-swapped dimers of cystatin C and stefin A had been determined by crystallography and NMR (nuclear magnetic resonance), respectively (Janowski et al., 2001; Staniforth et al., 2001). After solving the tetramer structure, we suggested that tetramers could be direct building blocks of amyloid fibrils, but the fibrillation reaction proved to be not that straightforward. It is more likely that such a tetramer is off-pathway, preventing fibril formation at higher protein concentrations (Škerget et al., 2009). A molecular dynamics study by Zganec et al. (2015), reproduced as Figure 2, indicated that some of the oligomers may be functional, exerting amateur chaperone and redox-sensitive functions, and in such a way may protect neurons from neurodegeneration. However, other types of oligomers may exert toxic functions by perforating membranes.

### Mechanism of amyloid fibril formation

**Figure 3:**
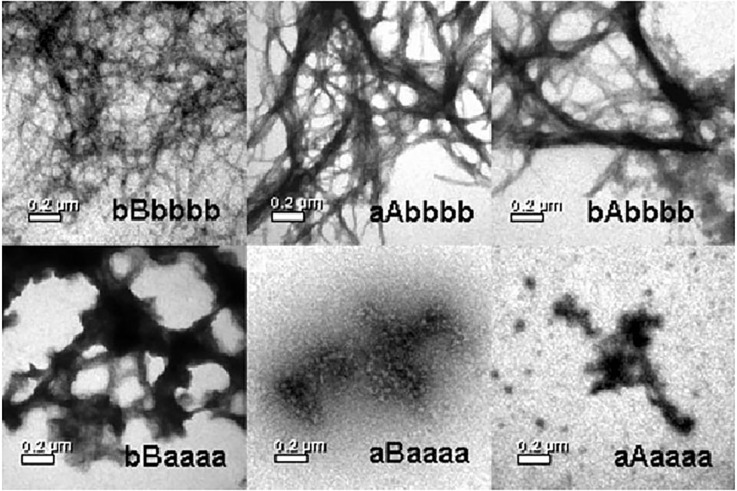
Propensity of the chimeras to form amyloid fibrils (in bold, below). TEM images of **stefin B (bBbbbb)**, the chimeric proteins **bAbbbb, aAbbbb**, bBaaaa, aBaaaa, and stefin A (aAaaaa) at the plateau stage of the amyloid fibril formation. Reproduced from Kenig et al. (2006).

Similarly to folding, a somewhat mixed fibril formation mechanism seems to apply in the case of stefin B—i.e., it is somewhere between a nucleated polymerization and conformational conversion on the nucleus, producing a long lag phase and a downhill polymerization without a lag phase. At pH 3.3, the reaction shows no lag phase and ends in necklace-like protofibrils from globular oligomers. At pH 4.8, a long lag phase applies (Žerovnik et al., 2002; Jenko et al., 2004). This is where domain-swapping most likely occurs, as the high energy of activation has to be surmounted (Škerget et al., 2009). A study of the chimeras was designed in order to investigate which parts of stefins dictate aggregation behavior (Kenig et al., 2006). It found that the propensity to form amyloid fibrils was in this order: **stefin B** > **bAbbbb** > **aAbbbb** > bBaaaa > aBaaaa > stefin A (where the lower case letters stand for β-strands and the capital letters for the α-helices of stefins A or B, respectively). This order does not correlate with stability, nor with the folding rates. Instead, the propensity to form amyloid fibrils is related to the β-sheet structure of stefin B. TEM results showing the morphology of amyloid fibrils and aggregates at the plateau of the fibrillation reaction are reproduced in Figure 3.

### Pore formation

We have shown that higher oligomers and prefibrillar oligomers of stefin B are indeed toxic to cells and that this is related to membrane perforation (Anderluh et al., 2005; Čeru and Žerovnik, 2008; Čeru et al., 2008). This is in accordance with other studies of pathological and model amyloidogenic proteins. For example, Kayed et al. (2020) studied oligomeric and multimeric species of α-synuclein in interaction with cellular membranes. Interaction of Aβ peptide with cellular membranes led to perforation for the more toxic Aβ (1–42) variant (Bode et al., 2017).

More precisely, we have shown that wild-type stefin B and missense EPM1 mutants (G4R, G50E) make pores into lipid bilayers, mimicking cellular membranes. Two types of pores were detected *in vitro* (by Rabzelj et al., 2008): smaller and more discrete currents were observed with the wild-type protein, while larger, less specific pores were observed with the prefibrillar states of this amyloidogenic protein, each of which can be obtained at pH 5 and 3, respectively.

At first glance, the structure and folding mechanism of a protein do not seem to have much in common with its function in normal cell physiology and dysfunction in disease. However, to understand stability, the folding and aggregation behavior of the wild-type protein in comparison to misfolded variants, under both normal and extreme conditions, is of utmost importance. It is often argued that EPM1 disease is not an amyloid disease. However, as will be shown later, a more severe phenotype might arise with stefin B (cystatin B) mutations and environmental factors, such as redox state, copper binding, and interaction of the oligomers with cellular membranes.

## The Functional Roles of Stefin B

### Knocking down the cystatin B gene produces most EPM1 symptoms; microglia activation, oxidative stress, and neuroinflammation

A representative animal model of EPM1 has been developed by knocking out stefin B gene in mice (stefin B KO mice). These mice develop key features of EPM1, including myoclonus, ataxia, and progressive neurodegeneration (Pennacchio et al., 1998; Tegelberg et al., 2012; Manninen et al., 2013). Early activation of the microglia precedes neuronal loss in the brains of stefin B KO mice (Tegelberg et al., 2012). The region most significantly affected is the cerebellum, where altered JAK-STAT pathway signaling (Körber et al., 2016; Rivera et al., 2016) leads to microglia activation and upregulation of genes associated with the immune system (Kälviäinen et al., 2008; Tegelberg et al., 2012). Pro-inflammatory chemokines and cytokines are also highly expressed in the stefin B KO mice model, thus lowering the seizure threshold and contributing to recurrent epilepsy (Devinsky et al., 2013). Maher et al. (2014a) reported that IFN-γ- and lipopolysaccharide (LPS)-activated stefin B-deficient macrophages produced higher amounts of NO and expressed more inducible nitric oxide synthase (iNOS) than wild-type macrophages. They also showed a decreased expression of interleukin (IL)-10 due to impaired STAT3 signaling (Maher et al., 2014a). Comprehensive reviews on the role of stefin B in the innate immune system and in neuroinflammation, respectively, have been written by Kopitar-Jerala (2015a,b). IL-10 has an essential role in mediating inflammatory processes, not only in the cells of the immune system but also in the brain (Zocchia et al., 1997). It has been demonstrated that IL-10 increases the survival of cerebellar granule cells by blocking caspase-3-like activity (Bachis et al., 2001). These findings are supported by Okuneva et al. (2015), whose study of cystatin B KO mice revealed higher levels of cystatin B (i.e., stefin B) mRNA expression in microglia than in neurons or astrocytes (Okuneva et al., 2015). Increased expression of pro-inflammatory iNOS, anti-inflammatory arginase 1 (ARG1), and chemokines was also observed (Okuneva et al., 2015).

Maher et al. (2014b) showed that upon LPS stimulation of activated macrophages, stefin B was targeted into the mitochondria, resulting in the enhanced destabilization of mitochondrial membrane potential and mitochondrial ROS generation by a mechanism that is still not yet completely clear. Indeed, the induction of ROS in the microglia of EPM1 patients due to loss of stefin B function might play a role in noncanonical inflammasome activation69 and cell death in the cerebellum of these patients. Manninen et al. (2014) showed that a progressive, albeit a non-uniform, volume loss occurs in the brain of stefin B KO mice, indicating that different neuronal populations possess different vulnerabilities to oxidative stress. They suggested that the white matter damage could be secondary to glial cell activation and neurodegeneration (Manninen et al., 2014).

### Impairment in synaptic transmission

As reported by Penna et al. (2019), stefin B plays an important role in the synapse. In accordance, Joensuu et al. (2014) found that in the cerebellum of pre-symptomatic stefin B KO mice, multiple changes in gene expression related to synapse maturation, development, and function were determined, the most prominent of which were changes in GABAergic signaling. It is known that GABA plays a central role in controlling neuronal development and connectivity; therefore, defective GABAergic signaling in the cerebellum of stefin B KO mice underlines ataxia in these mice (Grusser-Cornehls and Baurle, 2001). Gorski et al. (2020) observed a decreased abundance of sodium- and chloride-dependent GABA transporter 1 (GAT-1) using mass spectrometry (MS) data from cerebellar synaptosomes of presymptomatic stefin B KO mice. They also observed early mitochondrial dysfunction, which leads to altered synaptic function in EPM1 (Gorski et al., 2020).

Recently, Di Matteo et al. (2020) found that stefin B is essential for cell proliferation and migration during brain development. It influences the early stages of human neurogenesis by acting as a short- and long-distance mediator of the extracellular matrix. With such functions in the brain, stefin B may be linked to GABAergic (Franceschetti et al., 2007; Buzzi et al., 2012; Joensuu et al., 2014; Gorski et al., 2020) and serotonergic (Arbatova et al., 2005) neurotransmission.

### Stefin B wild-type and aggregation of selected EPM1 mutants in cells

Gains in toxic function and losses of function occur upon protein aggregation. To date, most of the aggregation studies of stefin B have been done *in vitro* (Rabzelj et al., 2005). However, in a study by Čeru et al. (2010b) and Polajnar et al. (2012, 2014a); we expressed wild-type (wt) stefin band some missense EPM1 mutants in mammalian cells and followed the aggregate morphology, as well as oxidative stress and cell death. Imaging by confocal microscopy of EPM1 mutants G50E and Q71P in comparison to the wt showed that the aggregates in cells are of two types: bigger perinuclear aggregates of G50E and Q71P (aggresome-like aggregates, as previously observed for R68X87); or smaller, more punctual, aggregates dispersed throughout the cytoplasm, which were observed for the wt and G4R. Somewhat surprisingly, the G50E and G4R aggregates proved more toxic, which could imply that mutants that aggregate quickly, such as R68X and Q71P, cause less damage than the aggregates of G50E, which aggregate slowly and form small cytotoxic oligomers, as is the case for G4R. Interestingly, overexpression of the wt protein also reduced cell viability (Polajnar et al., 2014a).

**Figure 4:**
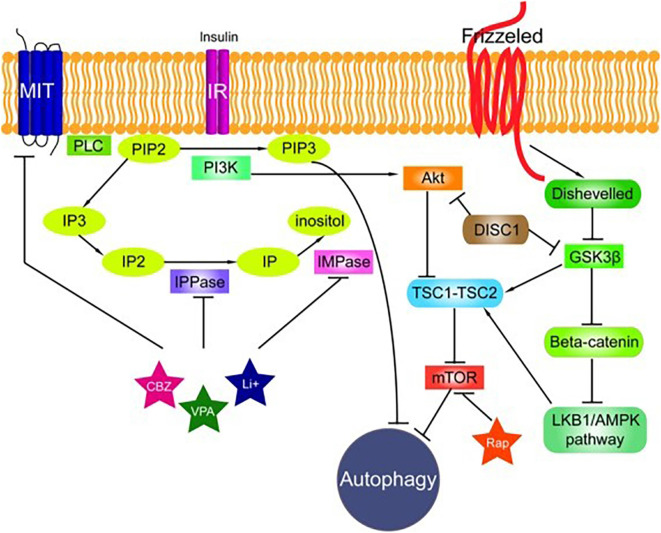
The signaling pathways connected to autophagy. The phosphatidylinositol signaling pathway is regulated by Class I phosphoinositide 3-kinases (PI3Ks), and PI3Ks are linked to an extraordinarily diverse group of cellular functions through regulation of the Akt/TSC1-TSC2/mTOR pathway. The Disrupted in Schizophrenia 1 (DISC1) gene negatively regulates both glycogen synthase kinase-3 beta (GSK3β) and Akt. Wnt signaling activation is mediated through the binding of a Wnt-protein ligand to a frizzled family receptor, which passes the biological signal to disheveled (Dsh). Dsh negatively regulates GSK3β, which alternatively inhibits β-catenin, one of the central proteins of the Wnt signaling pathway. Reproduced from Polajnar and Žerovnik (2014).

We have previously speculated on the alternative functions of stefin B (Škerget et al., 2010; Žerovnik, 2017a). In the study by Polajnar et al. (2014b), we showed that protein aggregates (of other proteins) increased upon knocking out the stefin B gene from the primary murine astrocytes. When monomers or small oligomers of stefin B were added to the cell medium, other protein aggregates decreased. Insoluble fractions of the aggregates were analyzed for protein composition using proteomics, and differences between wt and KO astrocytes were observed. Autophagic flux was also investigated, and it was found to be less efficient in KO cells. This is important, as autophagy is one of the main mechanisms for clearing cells of protein aggregates and deficient autophagy could lead to a buildup of the aggregated deposits. However, the mechanism of how stefin B influences autophagy remains to be clarified (Figure 4). Another option for how stefin B could influence proteostasis would be through a chaperone-like function. We have discussed alternative functions of stefin B before (Škerget et al., 2010; Taler-Verčič and Žerovnik, 2010; Žerovnik, 2017a). We proposed mutual chaperoning of amyloid proteins (Žerovnik, 2017b); for example, stefin B oligomers bind Aβ peptide (Škerget et al., 2010) and reduce its amyloid fibril formation.

## Strategies to Prevent Stefin B Aggregation and Possible Therapeutic Interventions

The finding by Rispoli et al. (2013) can explain the role of Cu^2+^ on stefin B (cystatin B) oligomerization. They reported that the monomeric protein requires Cu^2+^ for chaperone HSP70-dependent polymerization. They suggest that Cu^2+^ interacts with at least two conserved histidines, at positions 72 and 95, modifying the structure of native monomeric stefin B, which seems in accordance with our *in vitro* studies, where two strong binding sites were detected (Žerovnik et al., 2006). In their studies, Rispoli et al. (2013) found that the Cys at site 3 is the necessary residue, undergoing oxidation by Cu^2+^, which then triggers polymerization in conjunction with HSP70. In contrast to the polymers obtained from denatured stefin B monomers, the Cu^2+^/HSP70-dependent polymers are sensitive to reducing agents. It is thus likely that the polymers are physiological and functional. They may have alternative functions besides the anti-protease activity of monomeric stefin B.

One of these functions, as Rispoli et al. (2013) suggest, might be that the functional oligomers can sense the redox state of the cell and dissociate in the cytosol. If the cell’s environment would then become more acidic, with elevated ROS and in the presence of Cu^2+^/HSP70, stefin B would form oligomers and interact with partially folded or amyloid forming proteins, such as Aβ, acting as an amateur chaperone. This interaction of stefin B oligomers with Aβ was detected *in vitro* and in cells (Škerget et al., 2010; Žerovnik, 2017a). It would also be interesting to know if the more toxic form of stefin B oligomers could penetrate the mitochondrial membrane and cause leakage of ions and, finally, damage to mitochondria. Other recognized amateur chaperones (Wilhelmus et al., 2007) include small heat shock proteins, among them the crystallins.

We have studied the effect of polyphenols, vitamin C, and N-acetyl cysteine (NAC) on stefin B aggregation. In Hasanbašić et al. (2018), we reported on how polyphenols, vitamin C, and other antioxidants influence stefin B aggregation. It is well known that polyphenols such as curcumin often inhibit protein aggregation, which may explain their neuroprotective role. In another study (Jahić Mujkić et al., 2021) we observed a synergy of the inhibitory actions of vitamin C and some polyphenols. Synergy with vitamin C was also observed with curcumin and quercetin.

## Crosstalk of Stefin B and Other Genes in The Manifestation of EPM1

The behavior of other genes that cause EPM1-like symptoms may prove helpful in determining cellular pathways where stefin B functions might be involved. There are reports that mutations in PRICKLE1 (Bassuk et al., 2008), SCARB2 (Berkovic et al., 2008), and α-synuclein (Eberhardt and Topka, 2017) genes cause similar symptomatology as observed in EPM1, including myoclonus and ataxia.

We checked GeneCards, a human genome database, for putative signaling pathways of stefin B and found two major pathways: the innate immune system and neutrophil degranulation. Upon infection, neutrophils, the most abundant white blood cells, migrate towards the inflammatory site. Neutrophils contain granules filled with antimicrobial or proteolytic proteins. Neutrophils, when primed, actively secrete cytokines and inflammatory mediators. Additionally, they stimulate T cells by presenting antigens to the MHC II complex (Wright et al., 2010).

Next, we checked GeneCards for other genes causing similar pathology to EPM1. PRICKLE1 is connected to planar cell polarity (PCP) signaling and Wnt signaling, particularly to non-canonical Wnt signaling. This latter type of signaling activates beta-catenin-independent pathways that influence morphogenesis by negatively influencing the cytoskeleton (van Amerongen, 2012). Namely, PCP signaling is connected, at least in part, the actin cytoskeleton remodeling (Feng et al., 2021). A study of PRICKLE1 expression in the central nervous system (CNS) using *in situ* hybridization (ISH) and genetic knock-in methods showed that it has an important function in the development and maturation of the axonal network in neurons (Liu et al., 2013). This is significant in light of previous reports that cystatin B interacts with different partners involved in cytoskeleton formation (Cipollini et al., 2008; Rispoli et al., 2013). The same group also reported that cystatin B takes part in neural signal transmission in the synapse (Penna et al., 2019) and is essential for cell proliferation and migration during brain development (Di Matteo et al., 2020). They suggested that the polymeric nature of stefin B in cells could have a scaffolding role, similar to actin and tubulin (Rispoli et al., 2013).

SCARB-2 is connected to these two main pathways: cargo recognition in clathrin-mediated endocytosis and vesicle-mediated membrane transport. Vesicular transport pathways include vesicle formation, coating, budding, uncoating, and target membrane fusion. Vesicle-mediated transport takes place either from the cytosol *via* the endoplasmic reticulum (ER) and Golgi to the cell medium (exocytosis), or by transporting material from outside of the cell *via* scavenger receptors (endocytosis). Of note, vesicular transport was indicated in our differential proteomics study of the aggregated fraction of stefin B in KO astrocytes (Polajnar et al., 2014b).

α-Synuclein, whose mutations cause Parkinson’s disease (PD), has some parallels to stefin B, even though structurally they are quite different proteins. α-Synuclein is partly intrinsically disordered and partly α-helical, while stefin B is a globular protein with a 5-stranded β-sheet and one α-helix. Functionally, however, both proteins are involved in protein degradation pathways. α-Synuclein is connected to parkin-regulated ubiquitin proteasome degradation, while stefin B is connected to lysosomal degradation and autophagy. As described by Alakurtti et al. (2005), two missense mutants of stefin B, G4R and Q71P, fail to associate with lysosomes, implying an important lysosome-associated function for stefin B (Alakurtti et al., 2005). It should be recognized that the ubiquitin proteasome system (UPS) and autophagy are both connected to mitochondrial ROS production and mitophagy (Chan et al., 2011). The impairment of mitochondria is implied both in Parkinson’s disease and in EPM1 (Gorski et al., 2020). Notably, stefin B was found to be upregulated post-seizure in animal studies (D’Amato et al., 2000). Similarly, an increase in α-synuclein concentration was found (in the serum and cerebrospinal fluid) in epileptic patients as compared to normal controls (Rong et al., 2015). Even more interestingly, the level of α-synuclein was upregulated in patients with intractable epilepsy, while no association was observed in patients with non-intractable epilepsy (Rong et al., 2015).

In addition, both proteins, when undergoing transformation into the amyloid state, can make pores into membranes *via* the annular prefibrillar oligomers (Kim et al., 2009; Anderluh and Žerovnik, 2012; Tsigelny et al., 2012), which could lead to an imbalance of ions and thus have implications for neurodegeneration and epilepsy (Surguchov et al., 2017).

## Discussion and Future Perspectives

Traits common to neurological diseases accompanied by movement disorders—namely, epilepsy, ataxia, and neurodegeneration—are found in progressive myoclonus epilepsies (PMEs), Parkinson’s disease (PD), and Alzheimer’s disease (AD) and other dementias, as well as major psychiatric disorders, such as schizophrenia and major depression. Most of them are accompanied by chronic inflammation and glial cell activation. Meanwhile, most inherited mono gene dementias are accompanied by an increase in protein aggregates of the mutated gene or protein. Even sporadic AD or PD, which are multifactorial, are believed to begin from the toxic effect of intracellular protein aggregates, which most likely leads to an increase in oxidative stress by perforating membranes (though not necessarily in this order, as oxidative stress damage may make proteins more prone to aggregation). Protein aggregation has also been detected in models of schizophrenia and major depression; therefore, in papers by Polajnar and Žerovnik (2011, 2014); we suggested that impaired autophagy (Figure 4)—a likely consequence of incorrect protein folding and overwhelming aggregation to amyloid fibrils—could be a common trait, and a possible target, in PMEs, neurodegenerative diseases (with AD as a prototype), and neuropsychiatric diseases.

One can ponder if all these pathologies may not have certain upstream events in common, such as **protein misfolding**, **aggregation**, **and pore formation** (see Table 1). Together, these would trigger initial events [e.g., mitochondrial dysfunction with calcium influx (Pannuzzo, 2022), oxidative stress] followed by somewhat downstream endosomal/lysosomal dysfunction (Lee et al., 2022) and, *via* feedback loops, lead to greater accumulation of protein aggregates and perforation of membranes (leading again to more mitochondrial ROS and systemic inflammation). However, a counter-argument to such a hypothesis is that no protein aggregates, or amyloid fibrils, have been detected in EPM1 patient cells, whether from neurons or glia, or animal models. Another usual counter-argument is that stefin B is downregulated or nearly deficient in the majority of EPM1 cases, therefore a lack of its functions, not a potential gain in toxic function, must be important. However, many different mutations apart from dodecamer repeats have been observed, and these result in more aggregate prone proteins and may additionally complicate EPM1 pathology. Even in the case of dodecamer repeats and a lack of stefin B expression, if its functions are indeed connected to proteostasis, the cytoskeleton, and vesicular transport, impairment of such functions may lead to the aggregation of other proteins. These findings prompt us to take a closer and more careful look to reveal how protein aggregation in cells, neurons, and glia is associated with EPM1 pathogenesis.

**Table 1:** Development of EPM1 pathology from stefin B loss and gain of functions; central role of protein misfolding and aggregation.

1 **Protein misfolding and aggregation**. Protein aggregates (of other proteins) are not cleared, and they accumulate. Here, the possible **amateur chaperone function** of the functional oligomeric stefin B could play a protective role. It also can happen that stefin B itself and some EPM1 mutants form **toxic aggregates** and act on membranes as toxins (Pollard et al., 1993). Downstream, the **loss of function** of wt stefin B (by dodecamer repeats or aggregation) can influence lysosomes and autophagy. 2 **Dysfunction in lysosomal function**. Loss of the stefin B inhibitory function in the regulation of the cleavages by cathepsins; stalled autophagosomes. Impaired lysosomal function and impaired autophagy are reported for AD (Orr and Oddo, 2013; Lai et al., 2021; Lee et al., 2022). 3 **Dysfunctional autophagic flux** (Polajnar et al., 2014b). 4 **Loss of mitochondrial membrane integrity** (shown in LPS-stimulated primary bone marrow–derived macrophages from *Cstb^−/–^* mice; Maher et al., 2014b; Trstenjak Prebanda et al., 2019). Similar observations have been reported for AD (Vassallo, 2021) and PD (Ghio et al., 2019). 5 **Mitochondrial ROS** modulate GABAergic signaling in a cell-type-specific manner (Accardi et al., 2014). 6 **Increased oxidative stress**; decreased activity of SOD1, ROMO1, and the hemoglobin subunits HBA and HBB, linked to superoxide production. 7 **Neurodegeneration**: cerebellar granule neurons are especially sensitive to oxidative stress–induced cell death, with increased lipid peroxidation and depletion of antioxidants, in the cerebellum of *Cstb^−/–^*mice. 8 Altered abundance of key transport proteins, suggesting **altered axonal transport** in neurons. 9 Dysfunctions in global axonal transport may affect **synaptic plasticity**.

## Author Contributions

The author confirms being the sole contributor of this work and has approved it for publication.

## Funding

This work was financed by the J7-4050 project (EŽ) and by the program *P1-0140 proteolysis and its regulation* (led by Boris Turk, with an initial program by Vito Turk) obtained by the Slovenian Research Agency (ARRS).
